# Self-compassion as a protective factor against adverse consequences of social media use: A scoping review

**DOI:** 10.1371/journal.pone.0322227

**Published:** 2025-05-21

**Authors:** Deepa Manjanatha, Nicole Pippard, Cinnamon S. Bloss

**Affiliations:** 1 San Diego State University/University of California, San Diego Joint Doctoral Program in Clinical Psychology, San Diego, California, United States of America; 2 Center for Empathy and Technology, Institute for Empathy and Compassion, University of California San Diego, La Jolla, California, United States of America; 3 San Diego State University/University of California, San Diego Joint Doctoral Program in Public Health, San Diego, California, United States of America; 4 Herbert Wertheim School of Public Health and Longevity Science, University of California San Diego, La Jolla, California, United States of America; Universita Telematica Pegaso, ITALY

## Abstract

Self-compassion has been shown to foster wellbeing and act as a protective factor against the development of psychopathology. Its protective capacity against adverse consequences of social media use, however, is not well understood. Given the increasing use of social media in our daily lives, gaining a nuanced understanding of the relationship between self-compassion and adverse consequences of social media use may be important for building and maintaining healthier online behaviors and spaces. We conducted a systematic scoping review of empirical studies on the relationship between social media use, self-compassion, and wellbeing. We restricted our search to peer-reviewed studies published between January 2010 and October 2024 that were written or translated to English. Thirty studies of 32 independent samples including 11,991 participants were eligible for review. Many of these studies were cross-sectional surveys with majority female participants. Studies generally focused on one of two domains: 1) psychological well-being (e.g., anxiety/depression and resilience); and 2) body image and appearance (e.g., appearance dissatisfaction and comparison). The studies included in this review showed mixed results, with four common themes emerging: 1) There are associations between self-compassion and social media in a variety of populations and contexts; 2) Self-compassion may offer protective benefits in the context of social media use, but the extent and mechanisms remain unclear; 3) Self-compassion-oriented interventions may promote favorable health outcomes, with social-media-based approaches emerging as a promising avenue; and 4) Social media use is measured inconsistently, suggesting a standardized measure, while needed, is lacking. This review revealed a promising role for self-compassion in promoting wellbeing and buffering against the adverse effects of social media usage, while highlighting gaps and limitations of existing research. Future research should prioritize improvement in measurement of social media use and explore individual-level risk and resilience factors in the context of social media exposures and engagement.

## Introduction

Compassion, as understood by modern Western society, can be defined as being aware of others’ emotions and experiences, namely distress and sorrow, accompanied with the desire to help or comfort that person [1–3]. Self-compassion, on the other hand, has been defined as turning this compassion inwards when experiencing struggles or difficulties, by extending self-kindness, contextualizing one's struggles in the broader context of humanity, and being mindful of what one is experiencing without over identifying with negative thoughts or emotions [4]. Self-compassion is a construct originating in Eastern philosophical thought, most notably in Buddhist teachings, and is theorized as distinct from constructs such as self-centeredness or self-esteem because it is a self-regard construct that encompasses the concept of compassion [4]. More specifically, self-compassion centers how someone feels about themselves beyond outside influences, while self-esteem focuses on how an individual sees themselves in the context of others’ opinions [5].

### Conceptualization and measurement of self-compassion

The Self-Compassion Scale (SCS), developed by Kristin Neff in 2003 [4], was the first instrument in Western psychology to measure self-compassion. Despite recent development of additional scales to measure this construct, such as the Compassionate Engagement and Action Scales for Youths (CEASY) [6] and the Sussex-Oxford Compassion Scale-for the Self (SOCS-S) [7], Neff’s SCS is still the most widely used self-compassion measure. The SCS conceptualizes self-compassion with three basic components: 1) extending kindness to oneself instead of self-criticism or judgment; 2) seeing individual experiences as part of a broader human experience rather than isolating; and 3) maintaining balanced awareness when holding painful thoughts and feelings, without over-identifying with them. The original long form Self-Compassion Scale was developed in 2003 and is comprised of 26 items spread across six subscales (Self-Kindness, SK; Self-Judgement, SJ; Common Humanity, CH; Isolation, I; Mindfulness, M; Over-Identification, OI), with responses provided using a 1–5 Likert-type scale [4]. Since the development of this original scale, numerous adapted and translated versions of the scale have been developed and validated, including the widely used Self-Compassion Scale- Short Form (SCS-SF) which was developed and validated in 2011 [8]. The SCS-SF is composed of 12 items spread across the six dimensions with two items corresponding with each dimension [8]. Neff has advocated for the SCS possessing a “higher-order model” structure, suggesting that reporting and use of a single total self-compassion score and six separate subscale scores would be appropriate [8]. However, various studies in subsequent years have debated what factor structure appropriately corresponds with this scale, with findings spanning across a variety of factor structures and impacting how scores from the measure may be interpreted [9–11]. Neff’s self-compassion measure emphasizes the application of compassionate behaviors or sentiments towards oneself and others. Neff has posited that self-compassion not only encompasses individuals’ ability to apply compassionate behaviors towards oneself but also their ability to do these things for others as well [4]. Furthermore, there has also been some discussion in recent literature about a possible distinction between trait and state self-compassion [12]. Where relevant, we discuss this distinction in our review.

### The unique essence and protective role of self-compassion

Neff’s justification for developing the SCS originated in her theory that the construct of self-compassion likely functions as a health asset and therefore possesses a unique essence that is distinct from self-centeredness or self-esteem [4,5]. Moreover, traditional self-regard constructs such as self-esteem may not be associated with the same protective benefits as self-compassion. For example, high self-esteem has been found to be associated with lower resilience [13] and greater reactivity to negative situations, while self-compassion may protect against the impact of these negative situations [14,15]. Neff [5] and Neff and Vonk [15], purport that when “accounting for the overlap between the two constructs, the variance accounted for by self-esteem reflects positivity of self-representations, whereas what is accounted for by self-compassion reflects acceptance of oneself” [16]. Neff posits that health assets like self-compassion protect against adverse outcomes from psychopathology and support and promote positive emotional management and coping practices in the face of adversity, and subsequent studies have provided support for many of these claims [17–19]. Neff and colleagues assert that if we can effectively measure self-compassion, we may be able to better understand it and more effectively foster it, allowing us to strengthen resilience and improve health and wellbeing among individuals [4,5].

In the years since the development of the SCS, several studies have established self-compassion as a predictor of positive wellbeing [18] and a protective factor mitigating adverse outcomes of psychopathology (e.g., suicidal ideation, self-harm, depressive symptoms, anxiety, and stress) [17,19]. Furthermore, self-compassion has also been found to be an important aspect of healthy development. Studies have shown that fostering and maintaining a healthy level of self-compassion from a young age can improve functioning, maturity, wellbeing, and connectedness, as well as stave off depression and anxiety [14,20]. High self-compassion has also been associated with high levels of resilience in both adolescents and adults [21], as well as enhanced curiosity [22]. As such, self-compassion is an important potential target for interventions aimed toward improving wellbeing and decreasing illbeing across both youth and adults.

### Health outcomes and social media use

Social media platforms have become one of the primary ways that people leverage the internet to build connection and community. Use of these platforms (e.g., Facebook, Instagram, and others) has been shown to facilitate connection across individuals and groups [23] and increase visibility and solace for historically marginalized communities and individuals with marginalized identities [24]. Social media can also have harmful consequences, however, and may be associated with harmful effects for some individuals and groups based on identity and other vulnerabilities [25].

For instance, studies have shown evidence of harmful impacts of social media use, such as disturbance of body image, increased anxiety [26], increased depression [27], and increased suicide risk [28,29]. Moreover, problematic social media and/or smartphone usage patterns may present a wide variety of these types of harms to both adults [30] and youth [31]. In particular, excessive smartphone use has been found to be associated with depression, anxiety, OCD, ADHD and alcohol use disorders, in addition to difficulties with cognitive-emotion regulation, impaired cognitive function, and low self-esteem among adults [31]. Relatedly, a 2020 meta-analysis found a moderately strong association between problematic social media use and depression and loneliness [32]. Studies of social media usage in teens and adolescents also have highlighted detrimental impacts of frequent social media use on health risk behaviors such as alcohol use, substance use, sexual risk behaviors, and unhealthy diet behaviors [33]. Furthermore, the Surgeon General’s public health advisory on social media issued in May 2023 [34] highlights numerous harms to youth, for example, citing the longitudinal finding that spending more than three hours per day on social media doubles the risk of adolescents experiencing poor mental health outcomes such as depression and anxiety [34].

In addition to the unique harms to youth highlighted in the Surgeon General’s public health advisory [34], prior studies have also highlighted potentially disproportionate impacts of social media use on girls and young women [35]. One study deemed teenage girls as the “new high-risk group” [36] due to findings of worse outcomes compared to boys in terms of associations between social media usage and experience of negative feelings, low self-concept, low life satisfaction, and greater propensity for self-harm behaviors. These studies suggest that social media exacerbates existing risk factors in our society that uniquely threaten the wellbeing of young girls [37]. More broadly, studies of social media usage in teens and adolescents have highlighted detrimental impacts of certain use patterns on sleep, physical activity, anxiety, and depression [35,38].

### Self-compassion and social media use

Self-compassion has been studied and proposed to protect vulnerable social media users from the effects of anxiety and depression [39–41], but there has not been a definitive consensus on how this may operate. Challenges related to measurement of social media use and methodological limitations (i.e., largely correlational studies) contribute to this lack of consensus [25,42]. Given the integration of social media use in the lives of individuals, designing studies to test causal impacts of social media use on wellbeing and self-compassion have proven challenging. Studies examining the buffering potential of self-compassion may be inconclusive due to lack of uniformity in the measurement of social media use [39–41] and have often looked at a wide variety of health outcomes and contexts. These studies have also not been as widespread as those examining self-regard constructs such as self-esteem. In addition, no prior study has examined the landscape of literature looking at associations between self-compassion, social media, and wellbeing. As such, findings from studies examining mental health, patterns of social media use, and proximal constructs, such as self-esteem, may offer useful theoretical insights for hypothesizing how self-compassion may operate as a protective factor.

For instance, one study [43] found that higher levels of emotional problems and social media addiction in adolescents was associated with lower self-esteem and that social media addiction partially mediated the association between emotional problems and self-esteem. Though the study was cross-sectional, results support the notion that adolescents with higher levels of emotional problems tend to report higher levels of social media addiction and that there is also a relationship to self-esteem [43]. However, self-compassion is a different construct than self-esteem, as self-esteem measures only assess how an individual sees themselves in the context of others’ opinions while Neff’s self-compassion measure aims to assess how someone feels about themselves beyond outside influences [4]. Based on this distinction, self-compassion may function as a more robust protective factor against the adverse effects of social media usage, warranting further investigation.

Though studies examining related self-regard constructs such as self-esteem offer useful insights, the ways in which self-compassion may relate to and interact with social media use to influence health are not yet well understood. Additionally, the factors relevant to understanding this interplay have not yet been identified or explored, which is an aim of this scoping review. Applying a positive health perspective, where health assets rather than deficits are studied [44], has the unique potential to provide us with the tools we need to build healthier online spaces that highlight the ‘good’ while mitigating the ‘bad.’ As such, conceptualizing how self-compassion interfaces with social media use may lead to significant breakthroughs in building positive online experiences and understanding varying impacts of health assets on mental health and wellbeing more broadly. To address these broad research needs, this review aims to provide the first detailed overview of existing literature on the association between self-compassion and adverse consequences of social media use.

## Materials and methods

### Search strategy

This review was conducted and reported using the PRISMA-ScR (Preferred Reporting Items for Systematic Reviews and Meta-Analyses) extension protocol for scoping reviews (S1 Checklist). We conducted a systematic scoping review using narrative synthesis to analyze existing evidence. A structured literature search was performed including studies published between January 2010 and October 2024. Our search was initially conducted October 2022 to December 2022 and then updated March 2023 to June 2023, and we used Google Scholar, PubMed, PsycInfo, and PsycArticle databases. We subsequently conducted an updated search in October 2024 (using the same databases as the original search) for any studies published in 2023 and onwards.

Beginning in 2010, social media became what it is today, transforming from what had previously been a way to connect with others with similar hobbies online to platforms for creating and sharing user-generated content more broadly [45]. Therefore, this timeframe was selected both to allow a focus on recent research and to capture the period when social media as it currently exists, came to fruition. Our search strategies (reference S1 Table for more detailed information about search terms and search strategy) were tailored for each database, using keywords and Medical Subject Headings related to social media usage (e.g., “social media use,” “social media usage,” “social media profile”) and self-compassion (e.g., “self-compassion,” “self compassion,” “self regard”).

The initial search, performed by one author (DM), retrieved 2,710 publications across the various search platforms. The second search, performed three months later by a different author (NP), sought to replicate the first search. The two searches yielded several discrepancies (e.g., number of articles produced for identical search terms), possibly due to changes made to the Google Scholar platform algorithm in the intermediate three months. As a result, both authors then conducted the search a third time, leveraging PubMed, PsycInfo, and PsycArticle databases, in addition to Google Scholar, and found that the results from the second search were replicated in this third search. Therefore, the results from the initial search were discarded, and those from the replicated search are the results presented in this paper. The updated follow-up search conducted in October 2024 (limited to studies published in 2023 onwards) retrieved 1,212 publications across the various search platforms. In total, there were 3,922 total publications retrieved across searches. After removing titles that were duplicates and/or were not peer-reviewed journal articles (i.e., theses, books or book sections, reports, and conference papers and abstracts), 354 titles were removed, and 3,568 total abstracts and titles were eligible for screening.

### Article selection

**Fig 1:**
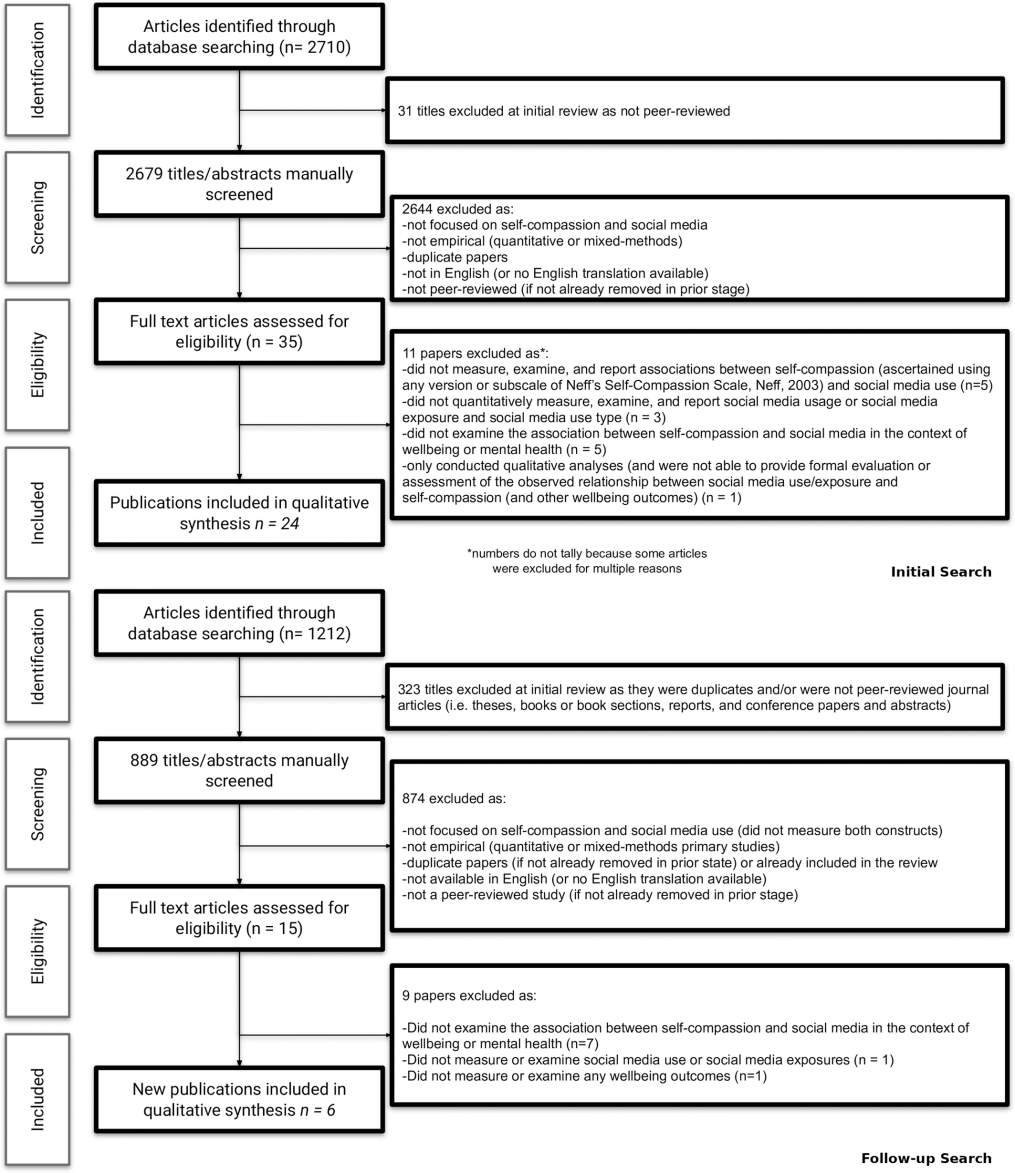
PRISMA Flowchart of article searching and exclusion.

After initial removal of duplicates and non peer-reviewed articles from the search results, a total of 3,568 abstracts and titles were eligible for screening (see Fig 1).

Two reviewers (DM and NP) manually screened these titles and abstracts, coding for inclusion or exclusion for full text review based on the following criteria: 1) Publication between January 2010 and October 2024, 2) Peer-reviewed, 3) Published in English or translated into English, 4) Examined or explored associations between any facet of social media use and self-compassion in the context of mental health or wellbeing (with measurement of these constructs). In our initial search we discovered that nearly all studies that met these criteria used either quantitative or mixed methods and measured self-compassion using any version or subscale of Neff’s Self-Compassion Scale [4]. Therefore, we adopted these latter two as additional criteria.

### Data extraction

Of the 3,568 titles and abstracts reviewed, we excluded 3,518 papers because they did not meet our eligibility criteria (described in the prior section) for full text review. In total, the 3,518 papers were removed for one or more of the following reasons: did not focus on or measure self-compassion and social media usage, were not empirical and primary studies (quantitative or mixed-methods), were duplicates that had not already been removed, were duplicate papers already included in the review, were not available in English and no version translated to English was available, or were not peer-reviewed (and were not already removed in a prior stage).

In total, the full text of the remaining 50 articles was assessed and an additional 20 articles were excluded for one or more of the following reasons: 1) article did not measure, examine, or report associations between self-compassion and social media use (n = 5); 2) article did not measure, analyze, or report social media use or social media exposure quantitatively (n = 4); 3) article did not examine the association between self-compassion and social media in the context of wellbeing or mental health (n = 12); 4) article did not examine or report any wellbeing outcomes (n = 1); and 5) article only conducted qualitative analyses and was not able to provide formal evaluation or assessment of the observed relationship between social media use/exposure, self-compassion, and other wellbeing outcomes (n = 1). Papers that may not have explicitly measured social media usage but did use different types or categories of social media experiences as exposures or covariates were included since these studies still evaluated associations between types of usage or exposures and self-compassion and/or wellbeing related outcomes. Papers reporting on the use of “simulated” social media use or controlled social media exposures (e.g., experimental, quasi-experimental designs) were eligible for inclusion if they provided concrete descriptions of social media related exposures used in the study. We did not limit our review to studies that used specific social media measurement tools, as there is a lack of consistency in how studies have operationalized the measurement of social media use [46]. Papers that only examined social media use and self-regard constructs such as self-esteem, without inclusion of the self-compassion construct, were excluded from the review. Papers that only examined social media use and psychopathology without any mention or inclusion of self-compassion were also excluded from the review. Papers that explored social media use, self-compassion, and aspects of psychopathology, but did not explicitly examine the associations between social media use and self-compassion were excluded. In total, 30 papers (each corresponding to a unique study) satisfied eligibility criteria and were included in the full text review (Fig 1). There were no geographical restrictions for inclusion in the review. In all, 30 publications comprising 32 samples satisfied our eligibility criteria (Fig 1).

For each study, the two reviewers (DM, NP) independently extracted data on the following dimensions: 1) year of study, 2) country where the study was conducted, 3) study design, 4) characteristics of the study population (sample size, research setting, and participant age, gender, language, and nationality) or target population (if applicable), 5) exposures/predictors (and how they were measured), 6) covariates (and how they were measured), 7) outcomes (and how they were measured), and 8) main findings. In addition, the two reviewers also provided an assessment of each study’s 9) strengths and limitations and 10) main implications.

### Analysis

Given the heterogeneity in study design of included papers, a narrative approach was used to summarize and present findings from the systematic scoping review process. Dimensions described as part of the data extraction process were used to guide the development of themes most relevant to addressing the overarching research questions.

## Results

### Overview of findings

A total of 30 manuscripts involving 32 study samples published between 2010 and October 2024 were eligible for this review. Despite searching for studies within this timeframe, the first study eligible for inclusion in our review was not published until 2016. Two of the 30 papers reported on two studies each. For the purpose of this review, we will be treating these studies as one manuscript but reporting on sample characteristics and the results of the embedded studies separately. Of the studies included in this review, 19 were cross-sectional quantitative studies and 11 were quasi-experimental, consisting of 10 intervention studies and one Ecological Momentary Assessment (EMA) measurement study. The majority of studies used online recruitment and online surveys (n = 19). In addition, n = 6 used in-person recruitment and in-person surveys, n = 1 used online recruitment and in-person surveys, n = 3 used in-person recruitment and online surveys, and n = 1 used a combination of in-person and online recruitment and online surveys. Of the 30 manuscripts, more than half were set in academic settings: within universities (n = 14), secondary schools (n = 2), and elementary schools (n = 1). See Table 1 for a summary of these study characteristics.

**Table 1:** Study Characteristics of Twenty-Four Included Articles on the Association Between Social Media Use and Self-Compassion.

Article (year)	Study Characteristics				
**Study Design**	**Study Setting** ^a^	**Role of Self-Compassion**	**Outcomes**	**Key Findings**
Adkins et al. (2023) [47]	Cross-Sectional Survey	United Kingdom; Recruited & surveyed online	Moderator	Internalized stigma	Self-compassion did not moderate the relationship between social media use and stigma. Self-compassion was negatively correlated with upward appearance comparisons and feelings of stigmatization.
Arigo et al. (2021) [48]	Quasi-Experimental Intervention	United States; Recruited in-person & surveyed online	Exposure via intervention	Body Satisfaction, Exercise Motivation, & Exercise Behavior	Expected benefits of self-compassion messages for body satisfaction and exercise were not observed. Results suggest that self-compassion messaging may be optimal for promoting positive outcomes in men and images without text may be optimal.
Barron et al. (2021) [49]	Quasi-Experimental Intervention	United States; Recruited & surveyed online	Exposure via intervention & Outcome	Self-Compassion, Body Satisfaction, Body Appreciation, & Appearance Comparison	Self-compassion group reported higher body satisfaction than fitspiration and control groups. Fitspiration images alone promoted lower body satisfaction and appreciation compared to the combined condition (fitspiration and self-compassion group).
Barry et al. (2020) [50]	Cross-Sectional Survey	United States; Recruited & surveyed online	Outcome	Self-Compassion, Loneliness, Self-Esteem, Life Satisfaction, & Sleep Difficulties	Those who reported greater FoMo (overall, with family, and with friends) also reported lower levels of self-compassion across all age cohorts. Negative components of self-compassion (overidentification, isolation, and self-judgment) were positively related to FoMo while the positive components of self-compassion were not significant.
Boonlue et al. (2016) [51]	Cross-Sectional Survey	Thailand; Recruited & surveyed in-person	Outcome	Self-Compassion & Psychological Resilience	Those who sacrificed time with loved ones to spend more time on social media reported less self-compassion. Gender, degree, performance compared to peers, and teacher support significantly predicted self-compassion.
Burkauskas et al. (2022) [52]	Cross-Sectional Survey	International; Recruited & surveyed online	Outcome	Self-Compassion, Appearance Anxiety, Exercising, & Use of Performance-Enhancing Drugs	Self-compassion was negatively associated with higher levels of time wasting and streaming internet behaviors.
Couto et al. (2023) [53]	Quasi-Experimental Intervention	United States; Recruited & surveyed online	Outcome	Self-Compassion, Trait Body Appreciation, State Body Appreciation, Self-Esteem	Interaction between presence/absence of body appreciation and objectification significantly influenced self-compassion, suggesting that self-compassion is higher in the presence of body appreciation messaging. No main effects found for body objectifying images or body appreciation images alone - Only the two presented together had an effect. Findings suggest body appreciation captions may serve as protective factors for self-compassion when young women view body objectifying posts (e.g., fitspiration) on SM.
Einstein et al. (2023) [54]	Cross-Sectional Survey	Australia; Recruited in-person & surveyed online	Moderator	Anxiety	Self-compassion was not a significant moderator in the relationship between social media frequency and anxiety after controlling for gender and age.
Feizollahi et al. (2021) [55]	Cross-Sectional Survey	Iran; Recruited & surveyed online	Predictor	Symptoms of Psychosomatic Disorders	Self-compassion was significantly related to smart phone addiction. The relationship between self-compassion and symptoms of psychosomatic disorders was fully mediated by smart phone addiction.
Gobin et al. (2022) [39]	Quasi-Experimental Intervention	United States and Canada; Recruited & surveyed online	Exposure via intervention	Body Image, Weight & Appearance Dissatisfaction	Self-compassion intervention led to decreased weight and appearance dissatisfaction after Instagram use while the control group showed an increase in body image concern after use.
Gracias et al. (2024) [56]	Cross-sectional Survey	United States; Recruited & surveyed online	Outcome	Self-Compassion, Disordered Eating, Levels of Weight/Shape Concerns	Self-compassion was significantly lower in the intentionally and incidentally exposed to fitspiration groups compared to the unexposed group, suggesting that regardless of the intention fitspiration may be problematic.
Griffioen et al. (2023) [57]	Quasi-Experimental Intervention	Netherlands; Recruited online & surveyed in-person	Moderator	Affective Wellbeing & Positive Affect	Self-compassion did not significantly moderate the effects between social media use and wellbeing outcomes.
Guo et al. (2024) [58]	Cross-Sectional Survey	China; Recruited & surveyed in-person	Moderator	Depression, Self-Esteem	Self-compassion moderated the relationship between SNS use and self-esteem. Children with low self-compassion experienced a decrease in self-esteem if they reported greater SNS use (negatively associated), whereas children with high self-compassion experienced a positive effect of SNS use on self-esteem (marginally significant). Self-compassion did not significantly moderate the relationship between SNS use and depression. Overall, self-compassion moderated the indirect effect of SNS use on depression by way of self-esteem.
Keutler et al. (2022) [59]	Cross-Sectional Survey	Ireland; Recruited & surveyed online	Mediator	Subjective Wellbeing	Self-compassion was found to be a significant mediator of the association between perfectionistic self-presentation on social media and well-being.
Keyte et al. (2021) [40]	Cross-Sectional Survey	United Kingdom; Recruited & surveyed online	Predictor	Instagram intensity, Wellbeing, Depression, Anxiety, & Stress	Self-compassion had a significant direct effect on Instagram intensity and a significant indirect effect on intensity via well-being. Self-compassion was negatively associated with depression, anxiety, and stress.
Lonergan et al. (2019) [60]	Cross-Sectional Survey	Australia; Recruited & surveyed online	Moderator	Body Dissatisfaction	Self-compassion did not significantly moderate the association between body dissatisfaction and social media variables (photo manipulation and investment in others’ responses to selfies).
Mahon et al. (2022) [61]	Quasi-Experimental Intervention	Ireland; Recruited & surveyed in-person	Exposure via intervention & Outcome	Self-Compassion, Social Media Comparison, Self-Criticism, & Body Image Perception	Boys reported significant increases in body satisfaction pre- to post-self-compassion intervention. This effect was sustained at 3-month follow-up. Girls reported significant improvements in body appreciation and reductions in self-criticism pre- to post-intervention.
Mitropoulou et al. (2022) [62]	Cross-Sectional Survey	Greece; Recruited & surveyed online	Predictor	Social Media Addiction	Self-compassion and social media addiction were negatively correlated.
Modica et al. (2019) [63]	Cross-Sectional Survey	United States; Recruited & surveyed online	Moderator	Body Esteem & Body Surveillance	Self-compassion was significantly related to body esteem and body surveillance. Self-compassion did not significantly moderate the relationship between Facebook appearance comparison and body esteem or body surveillance.
Mosanya et al. (2024) [64]	Cross-Sectional Survey	Poland; Recruited & surveyed online	Predictor & Mediator	Self-Esteem, Body Image	Among women, Instagram and TikTok usage was negatively associated with self-compassion. Among women, there was a significant direct and negative effect of social media addiction on self-compassion. In mediation analyses, self-compassion appeared to be a substantial buffer to the negative effect of social media addiction on self-esteem. Among men, there was no direct effect of social media addiction on self-esteem; therefore mediation by self-compassion was not possible. Both males and females showed an effect of social media addiction on body image, allowing for mediation analyses for both genders. There was also a direct and negative effect of social media addiction on self-compassion for both groups. In mediation analyses, there was a significant indirect effect of self-compassion (-) on body image for females. Self-compassion also mediated the relationship between social media addiction and body image among males.
Phillips et al. (2021) [41]	Cross-Sectional Survey	Australia; Recruited & surveyed online	Moderator	Depression & Anxiety	Self-compassion buffered the relationship between social media use profiles (problem users, disenchanted dabblers, moderate users, contented dabblers) and depression and anxiety. Highly self-compassionate problem users reported similar levels of depression and anxiety to other profiles groups.
Roberts et al. (2022) [65]	Quasi-Experimental Intervention	United States and Canada; Recruited in-person & surveyed online	Mediator	Self-Objectification, Body Surveillance, & Body Shame	Self-compassion significantly mediated the change in both body surveillance and body shame.
Rutter et al. (2024) [66]	Quasi-Experimental Intervention	United States; Recruited & surveyed online	Moderator & Outcome	Self-Compassion	State self-compassion was significantly lower in the fitspiration condition compared to the three other conditions (body positive photos, body positive quotes, landscape photos). This effect was significantly moderated by trait self-compassion. State self-compassion was significantly higher after viewing no-make up photos compared to the two other conditions (makeup face, landscape photos). Trait self-compassion did not significantly moderate this observed effect.
Sabik et al. (2020) [67]	Cross-Sectional Survey	United States; Recruited online and in-person & surveyed online	Outcome	Self-Kindness, Resilience, Depression, & Stress	Higher self-worth dependent on social media was a significant predictor of lower self-kindness. Women whose self-worth was dependent on social media also reported lower levels of resilience and higher rates of stress and depression symptoms.
Seekis et al. (2020) [68]	Quasi-Experimental Intervention	Australia; Recruited & surveyed online	Exposure via intervention & Outcome	Self-Compassion, Upward Appearance Comparison, Social Appearance Anxiety, Body Dissatisfaction, Drive for Thinness, & Body Appreciation	Self-compassion intervention participants reported lower upward appearance comparison, social appearance anxiety, and drive for thinness post-intervention and at 3-month follow-up. These same participants also reported higher levels of body satisfaction and higher self-compassion post-intervention and at 3-month follow-up.
Seekis et al. (2023) [69]	Quasi-Experimental Intervention	Australia; Recruited & surveyed online	Exposure via intervention & Outcome	Self-Compassion, Face-related Appearance Shame, Anxiety, Mood, & Upward Appearance Comparisons	Self-compassion was lower within the beauty group relative to the self-compassion group and travel control, but higher in the self-compassion group relative to the travel control.
Shao et al. (2021) [70]	Cross-Sectional Survey	China; Recruited & surveyed online	Moderator	Emotion-Focused & Problem-Focused Outcome Reappraisal	Intrapersonal regulation, including self-kindness, generated a buffering effect on emotional exhaustion and promoted reappraisal toward a stressful situation. The benefits of interpersonal regulation were counteracted by social media and hyper-personal regulation.
Slater et al. (2017) [71]	Quasi-Experimental Intervention	United Kingdom; Recruited and surveyed in-person	Exposure via intervention & Outcome	Self-Compassion, Body Satisfaction, Body Appreciation, & Negative Mood	Self-compassion quotes improved self-compassion, body satisfaction, and body appreciation, and reduced negative mood. Lower levels of self-compassion were reported when women viewed fitspiration images compared to neutral images. Fitspiration images and self-compassion quotes combined led to more positive outcomes than fitspiration images alone.
Varaona et al. (2024) [72]	Cross-Sectional Survey	Spain; Recruited & surveyed online	Outcome	Self-Compassion, Self-Criticism, Body Dissatisfaction	No significant relationship was detected between Instagram usage time and levels of self-compassion. No significant associations were observed between self-compassion scores and daily Instagram usage or most-viewed content categories, aside from ‘humor’ content which had a negative association with self-compassion.
Wang et al. (2022) [29]	Cross-Sectional Survey	China; Recruited and surveyed in-person	Moderator	Body Dissatisfaction	Self-compassion moderated the indirect link between social networking sites body talk and body dissatisfaction via peer appearance pressure, indicating a potential protecting role of self-compassion in body image concerns.

a Including both country of origin and location of recruitment and surveillance (in-person vs. online)

### Participant characteristics

Data from 11,991 participants were analyzed across samples. Sample sizes ranged from 65 [65] to 2,223 [52] participants per study. Gender varied and included all female (12 studies), majority female (15 studies), and majority male (5 studies) samples. All studies reported age of participants, where mean age varied from 9.8 [58] to 35.9 [63] years, and age ranged from 8 [58] to 78 [70] years. Only half of the papers reported the race/ethnicity of their samples (n = 15). Of those that reported, all but one study [39] had majority White participants (>50% of the total sample). Although the Gobin article [39] reported less than fifty percent White participants, White was still the most represented race in the study. Additional races/ethnicities that were represented across study samples included: Black/African American, Hispanic/Latinx, Asian/Asian American/East Asian/South Asian, Middle Eastern, Native Hawaiian/Pacific Islander, American Indian/Alaskan Native, Aboriginal, African, and multiracial groups. Finally, studies were conducted across continents in countries including Australia (n = 5), Canada/America mix (n = 1), China (n = 3), Greece (n = 1), Iran (n = 1), Ireland (n = 2), the Netherlands (n = 1), Poland (n = 1), Spain (n = 1), Thailand (n = 1), the United Kingdom (UK) (n = 3), and the United States (US) (n = 9). One study contained a mixed international sample. Of the 30 manuscripts included in this review, none reported information on participants’ language, socioeconomic status, or nationality. See Table 2 for a summary of these sample characteristics.

Topic areas and themes/key findings of this review

**Table 2:** Sample Characteristics of Twenty-Four Included Articles on the Association Between Social Media Use and Self-Compassion.

Article (year)	Sample Characteristics			
	Total n	Age in years (mean, range)	Gender (%a)	Race (%a)
Adkins et al. (2023) [47]	650	24.5	Female (82.9%); Male (16.9%); Other (0.2%)	White (78.5%); Asian (14.4%); Mixed Race (4.0%); Black (1.5%); Arab (0.8%); Latin American (0.8%); Did not report (0.5%)
Arigo et al. (2021) [48]	655	18.9	Female (59.0%); Male (41.0%)	White (64.0%); Black (11.0%); Hispanic/Latinx (11.0%); Asian/Asian American (7.0%); More than one race (6.0%); American Indian/Alaskan Native (0.5%); Other (0.5%)
Barron et al. (2021) [49]	Study 1: 180 Study 2: 296	Study 1: 19.1, 18–33 Study 2: 26.8, 20–30	Study 1: Female (65.6%); Male (34.4%) Study 2: Female (41.6%); Male (58.4%)	Study 1: White (53.3%); Asian (21.2%); Black (9.4%); Hispanic/Latino (8.4%); Biracial (5.4%); Native Hawaiian/Pacific Islander (< 1%) Study 2: White (66.9%); Black (14.9%); Asian (10.1%); Hispanic/Latino (5.7%); American Indian/Alaskan Native (1.4%)
Barry et al. (2020) [50]	419	NA^b^	Female (75.9%); Male (23.4%); Did not report (0.7%)	White (72.5%); Black/African American (15.3%; Multiracial (4.1%); Hispanic/Latinx (3.3%); Other (2.3%); Asian (2.2%)
Boonlue et al. (2016) [51]	484	20.4, 17-44	Female (51.2%); Male (48.3%); Other (0.4%)	NA
Burkauskas et al. (2022) [52]	2223	33.0	Female (70.0%); Male (30.0%)	NA
Couto et al. (2023) [53]	200	19.8, 18-24	All female	NA
Einstein et al. (2023) [54]	951	13.7, 12-16	Female (46%); Male (54%)	NA
Feizollahi et al. (2021) [55]	254	20.1	Female (44.1%);Male (55.9%)	NA
Gobin et al. (2022) [39]	230	25.9, 18-55	All female	White (40.9%); Southeast Asian (21.3%); Middle Eastern (8.7%); Black (8.3%); East Asian (8.3%); Other (8.3%) Hispanic/Latinx (3.5%); Aboriginal (0.9%)
Gracias et al. (2024) [56]	234	19.9, 18-22	All female	White (82.1%); Asian/Asian American (8.1%); Bi/Multiracial (4.3%); Black or African American (2.6%); American Indian/Alaskan Native (0.4%); Other (2.1%)
Griffioen et al. (2023) [57]	117	20.8, 18-31	Female (98.3%); Gender Non-conforming (1.7%)	NA
Guo et al. (2024) [58]	386	9.8, 8-12	Female (42.5%); Male (57.5%)	NA
Keutler et al. (2022) [59]	129	23.6	Female (78.3%); Male (20.9%); Other (0.8%)	NA
Keyte et al. (2021) [40]	173	24.5	Female (66.5%); Male (32.9%); Other (0.6%)	White (83.8%); Asian (7.5%); Other (5.2%); Black (3.5%)
Lonergan et al. (2019) [60]	184	19.9, 17-40	Female (51.6%); Male (48.4%)	NA
Mahon et al. (2022) [61]	80	15.4	Female (67.5%); Male (32.5%)	White (98.8%); Black (1.3%)
Mitropoulou et al. (2022) [62]	255	27.0, 18-60	Female (69.0%); Male (31.0%)	NA
Modica et al. (2019) [63]	232	35.9, 20-72	All female	White (73.7%); Black (13.8%); Hispanic (4.7%); Asian (4.3%); Other (2.6%) American/Indian/Alaskan Native (0.9%)
Mosanya et al. (2024) [64]	527	25.2, 18-67	Female (67%); Male (30.6%); Did not report (2.4%)	NA
Phillips et al. (2021) [41]	300	34.7, 18-71	Female (77.3%); Male (22.7%)	NA
Roberts et al. (2022) [65]	65	14.3, 10-19	All female	White (80.0%); Hispanic/Latinx (14.0%); Asian American (7.0%)
Rutter et al. (2024) [66]	Study 1: 145 Study 2: 105	Study 1: 23.0, 19–26 Study 2: 26.8, 21–30	Study 1: All female Study 2: All female	Study 1: White (67.1%); Black (9.1%); Asian (8.4%); Multiple/Other (8.4%); Hispanic/Latina (7.0%) Study 2: White (66.7%); Asian (10.5%); Black (9.5%); Hispanic/Latina (6.7%); Multiple/Other (3.8%)
Sabik et al. (2020) [67]	164	20.5, 18-33	All female	White (79.9%); Multiracial (5.5%); Hispanic/Latina (4.9%); Asian/Pacific Islander (3.7%); African American (3.0%)
Seekis et al. (2020) [68]	76	18.0, 17-21	All female	White (75.0%); Asian (9.0%); Middle Eastern (8.0%); Pacific Islander (5.0%); Other (3.0%)
Seekis et al. (2023) [69]	115	19.4, 17-25	All female	White (70.4%); Asian (13.0%); Southeast Asian (5.3%); Other (5.2%); African (3.5%); Australian Aboriginal (1.7%); Middle Eastern (0.9%)
Shao et al. (2021) [70]	538	35, 18-78	Female (49.3%); Male (50.7%)	NA
Slater et al. (2017) [71]	160	21.2, 18-25	All female	White (50.3%); Asian (22.4%); Black (13.7%); Mixed (10.6%); Other (2.5%)
Varaona et al. (2024) [72]	1051	30.6, 18-50	Female (90.2%); Male (9.1%); Other/Did not report (0.7%)	NA
Wang et al. (2022) [29]	413	19.5, 16-22	All female	NA


^a^Percentages may not sum to 100 percent due to rounding; ^b^ NA = Not Available

### Topic area overview: psychological well-being and appearance/body image

Studies generally fell into two main topic areas: psychological well-being and appearance/body image. Psychological well-being studies focused on the examination of psychopathology (e.g., anxiety [41,54], depression [40,41,58,67]) positive psychological traits (e.g., well-being [40,57,59], positive affect [57]), and other related constructs (e.g., loneliness [50], sleep difficulties [50]) in the context of self-compassion and social media. Additional well-being-related constructs examined included: internalized stigma [47], self-esteem [50,58], life satisfaction [50], fear of missing out (FoMo) [50], resilience [51,67], symptoms of psychosomatic disorders [55], psychological flexibility [59], stress [40,67], emotion-focused outcome reappraisal [70], and negative mood [69,71]. Study designs varied from interventions with quasi-experimental designs to cross-sectional survey studies ascertaining exposures and outcomes at a single time point. One well-being-related study utilized the previously mentioned EMA measurement to collect data on participant social media behaviors in their natural environment in real time [57].

The second topic area, appearance/body image studies, focused on the examination of appearance perception (e.g., body dissatisfaction [29,60,72], appearance comparison [47,49,63,68,69]) and related behaviors (e.g., exercise activity [48,52], disordered eating [56]) in the context of self-compassion and social media. Additional appearance-related constructs examined included: feelings of stigmatization related to acne [47], body satisfaction [48,49,61,68,71], body appreciation [49,53,61,68,71], appearance anxiety [52], use of performance enhancing drugs [52], body image [39,64], weight and appearance dissatisfaction [39], levels of weight/shape concerns [56], social media comparison [61], body esteem [63], body surveillance [63,65], body shame [65], social appearance anxiety [68], drive for thinness [68], and face-related appearance shame [69]. Again, study designs varied from interventions with quasi-experimental designs to cross-sectional survey studies ascertaining exposures and outcomes at a single time point.

While a majority of studies examined either psychological well-being or appearance in relation to self-compassion and social media, eight studies [47,53,61,64,65,69,71,72] examined a combination of outcomes that spanned both topic realms. These studies examined psychological constructs related to stigma [47], self-esteem [53,64], self-criticism [61,72], self-objectification [65], and negative mood [69,71] in the context of appearance, making it challenging to disentangle the two topic areas.

**Fig 2:**
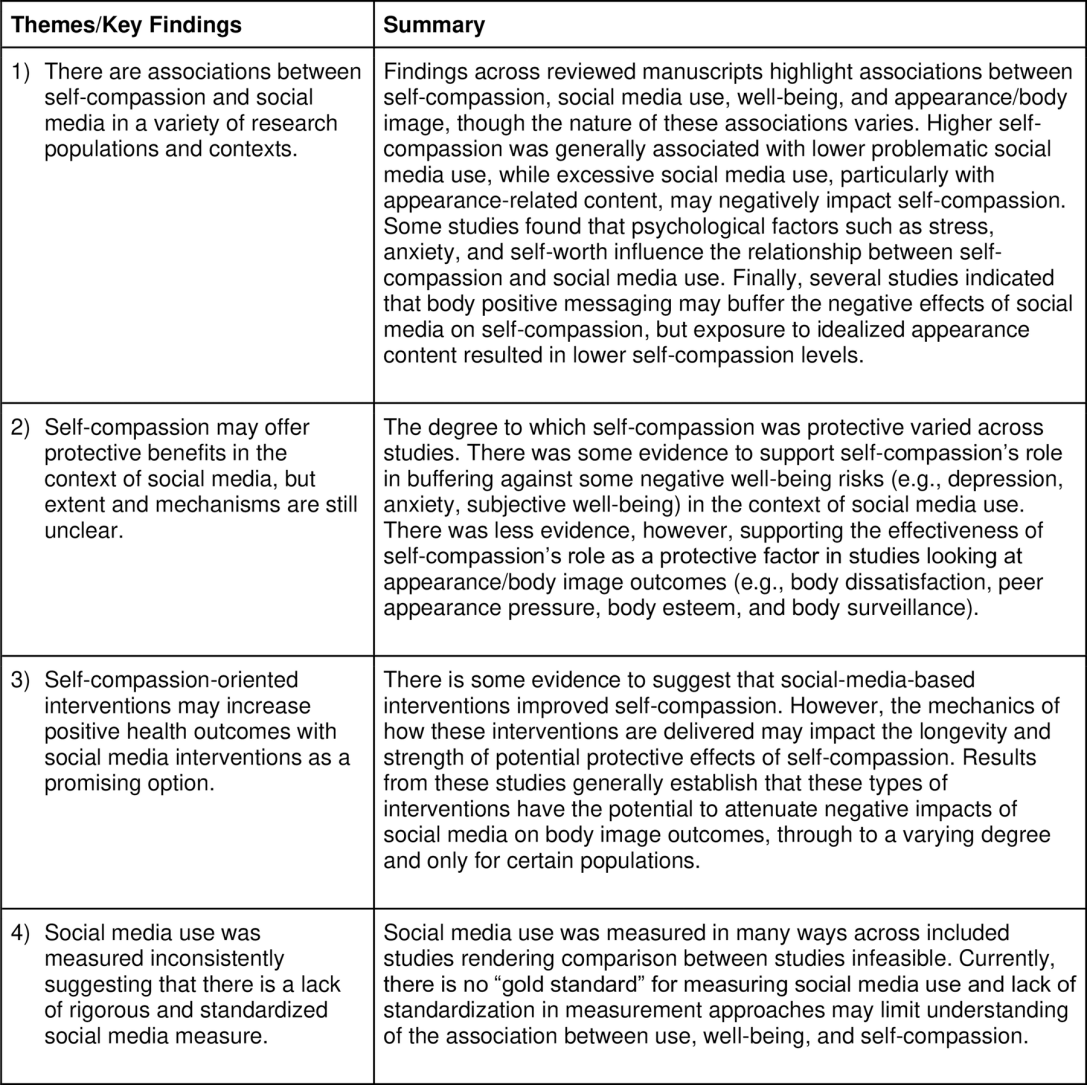
Themes/Key Findings.

Across both topic areas, self-compassion was examined as a predictor (4 studies), exposure (2 studies), mediator (3 studies), moderator (10 studies), outcome (8 studies), and both an exposure (via intervention or workshop) and outcome (5 studies). Furthermore, while all manuscripts included in our review measured self-compassion using adapted or derived versions of the Neff SCS [4], measurement of social media use varied widely in terms of general approach and specific tools that were used. Finally, four themes/key findings were identified based on examination of the 30 full-text articles and synthesis of findings: 1) There are associations between self-compassion and social media in a variety of research populations and contexts; 2) Self-compassion-oriented interventions may increase positive health outcomes including self-compassion, with social-media based interventions as a promising option; 3) Self-compassion may offer protective benefits in the context of social media, but extent and mechanisms are still unclear; and 4) Incongruent findings across the literature may be a result of lack of rigorous and standardized social media measurement among other study limitations. Fig 2 summarizes these themes/key findings.

#### Theme 1: There are associations between self-compassion and social media in a variety of research populations and contexts.

Findings across reviewed manuscripts demonstrated that there are associations between 1) self-compassion and social media use, 2) self-compassion and well-being constructs in the context of social media, and 3) self-compassion and appearance/body image constructs in the context of social media.

##### Self-compassion and social media use.

Several studies reported direct associations between social media use and self-compassion. Given the cross-sectional nature of a majority of the reviewed papers, this relationship was explored in both directions across studies where self-compassion was treated as both a predictor and outcome of social media use.

Three papers examined the direct effect of self-compassion on social media use in addition to other analyses [40,55,62]. A UK-based cross-sectional survey study conducted by Keyte and colleagues [40] found that self-compassion had a significant direct effect on Instagram Intensity - that is, those who reported higher levels of self-compassion also reported less time spent on Instagram. Similarly, Mitropoulou [62] examined the relationship between self-compassion and social media use among a sample of Greek adults and found that those with higher levels of self-compassion reported significantly less social media addictive behaviors. Finally, in an Iran-based study of university students, researchers [55] reported a direct and significant association between self-compassion and social media use where students who reported higher levels of self-compassion also reported lower levels of smartphone addiction. While it is encouraging that these findings are consistent, showing a significant relationship between self-compassion on social media use, it is important to note that all four studies were cross-sectional and conducted additional analyses to probe this relationship further. Specifically, mediation analyses of these cross-sectional survey studies will be further discussed in the next section of our results.

Looking at the reverse relationship, four studies examined the effect of social media use as a predictor of self-compassion [52,64,67,72]. A cross-sectional survey study conducted by Mosanya and colleagues [64] found that media addiction was negatively associated with self-compassion in Polish adults - that is, those with higher levels of media addiction reported significantly lower self-compassion. An international cross-sectional study that explored specific online behaviors and their association with psychological factors, including self-compassion [52] was also notable. Specifically, after adjusting for age, gender, country, and duration of internet use, Burkauskas and team found that higher self-compassion was negatively associated with increased internet time-wasting (time spent on applications that lack a specific benefit) and streaming, but not significantly associated with social networking. Varaona and colleagues [72] found similar results in a cross-sectional survey study based out of Spain. Contrary to their hypothesis, these researchers found that there was no significant relationship between time spent on Instagram and levels of self-compassion among Spanish adults aged 18–50. Another US-based cross-sectional survey study found a significant association between social media use and self-compassion but quantified both constructs differently [67]. Researchers examined the relationship between self-worth dependent on social media and self-kindness, one of the six dimensions of the SCS, and found that university women who reported higher self-worth based on social media also reported lower levels of self-kindness. These incongruent findings on the effect of social media use on self-compassion may be a result of measurement variation, which is further discussed below.

##### Self-compassion, social media use, and psychological well-being.

Twelve of the 30 papers reviewed examined associations between self-compassion, social media use, and psychological well-being. While many of these papers reported important findings regarding relationships between social media use and well-being outcomes, we report only on findings involving self-compassion in the context of well-being and social media use.

Of these twelve studies, three aforementioned articles examined self-compassion as a predictor of social media use and/or well-being in the context of social media use [40,55,62]. While direct and significant associations between self-compassion on social media use were previously reported in all three analyses, the inclusion of well-being measures allowed authors to further explore pathways to understanding these relationships. The previously mentioned Keyte article [40] found that the direct relationship between self-compassion and Instagram Intensity became nonsignificant with the addition of well-being, depression, anxiety, and stress variables on causal pathways. Specifically, these psychological variables added individually in four separate mediation models explain why or how self-compassion may influence Instagram Intensity by way of well-being, depression, anxiety and/or stress. Mitropoulou and colleagues [62] also discovered that the direct effect of self-compassion on social media addiction became nonsignificant when examining mediation through psychological distress, but interestingly only for positive dimensions of Neff’s SCS (mindfulness, common humanity, and self-kindness). Negative dimensions of self-compassion including self-judgment, isolation, and over-identification remained significantly correlated with social media addiction even with the addition of an explanatory pathway through psychological distress. Feizollahi and colleagues [55] found a direct and significant effect of self-compassion on smartphone addiction but examined a different pathway to understanding the relationship between the two in the context of well-being. The authors proposed a model in which smartphone addiction mediated the relationship between self-compassion and symptoms of psychosomatic disorders, where self-compassion was related to psychosomatic disorders by way of smartphone addiction. Results found smartphone addiction fully mediated the relationship between self-compassion and symptoms of psychosomatic disorder - that is, there was no direct relationship between self-compassion and symptoms with the introduction of smartphone addiction, suggesting that self-compassion impacted symptoms of psychosomatic disorders primarily through its influence on smartphone addiction. These findings further contribute to the understanding of the interplay of social media, self-compassion, and well-being.

Only two studies in our review examined self-compassion as an outcome in the context of social media use and psychological well-being [50,51]. Researchers from Thailand conducted a cross-sectional survey study among Thai undergraduate students to explore the effects of social media use on psychological well-being including self-compassion and psychological resilience [51]. Their study found that students who sacrificed time with friends and family to spend more time on social media were less self-compassionate and less resilient. Unlike other studies in this review, Boonlue and colleagues sought to understand predictors of self-compassion and reported that gender, degree, school performance in comparison to friends, friends outside the classroom, family support, and teacher support were associated with higher levels of self-compassion [51]. The article did not report on potential mechanisms for these associations. Finally, Barry and colleagues [50] hypothesized that Fear of Missing Out (FoMo) would be associated with lower self-compassion among a US sample of university students. In line with their hypothesis, researchers reported that those with greater overall FoMo, FoMo with family members, and FoMo with close friends also reported lower levels of self-compassion. Similar to findings from Mitropoulou’s aforementioned study [62], Barry and team [50] found that significant correlations between FoMo and self-compassion were only observed for negative dimensions (e.g., overidentification, isolation, self-judgement) of self-compassion.

##### Self-compassion, social media use, and appearance/body image.

Twelve of the 30 papers reviewed examined associations between self-compassion, social media use, and appearance/body image. While many of these papers reported important findings regarding relationships between social media use and appearance/body image outcomes, we report only on findings involving self-compassion in the context of appearance/body and social media.

Of these twelve studies, two examined self-compassion as a predictor of appearance/body image outcomes in the context of social media use [48,64]. Arigo and colleagues [48] aimed to examine the differential effects of fitspiration posts paired with fitspiration messaging, self-compassion messaging or no text (image only) in a group of US college students. Using a quasi-experimental design, researchers found no significant differences in body satisfaction or exercise motivation outcomes by exposure group. Conversely, in the aforementioned cross-sectional survey study, Mosanya and team [64] reported that self-compassion significantly predicted body image - that is, those with higher levels of self-compassion also had higher body image esteem.

Three studies in this review examined self-compassion as an outcome in the context of social media use and appearance/body image [53,56,66]. All three of these studies examined the effects of appearance-related content (e.g., fitspiration, makeup posts) on university-aged women in the US and found significant associations between social media and self-compassion. Couto and team [53] conducted a 2 (objectification presence vs. absence) x 2 (body appreciation presence vs. absence) online experiment where participants saw three Instagram posts manipulated to include objectification and/or appreciation content. They found that self-compassion was higher in the presence of body appreciation messaging, but only in the presence of objectifying images as well. These findings suggest that body appreciation messaging may serve as a protective factor when women view fitspiration posts, including those with objectifying content. In another study, Gracias and colleagues [56] asked participants about their interactions with appearance-related media and reported that intentional and incidental exposure to fitspiration posts on Instagram was associated with lower self-compassion in comparison to the no exposure group. Similarly, in the aforementioned Rutter article [66], researchers concluded that viewing appearance-related content had significant effects on state self-compassion. Those who were exposed to ‘appearance ideal’ posts (e.g., fitspiration or makeup faces) reported significantly lower state self-compassion post exposure compared to those exposed to body positive content (e.g., body positivity bodies, quotes, or no makeup faces) or appearance-neutral content (e.g., landscape photos). These findings from the Rutter article are particularly relevant as a quasi-experimental design was used to examine a change in state self-compassion before and after Instagram posts, allowing researchers to draw conclusions based on condition group and temporality.

#### Theme 2: Self-compassion may offer protective benefits in the context of social media, but the extent and mechanisms are still unclear.

Thirteen of the 30 papers in this review examined self-compassion as a mediator and/or moderator in the context of social media use, well-being and/or appearance/body image. A mediator is a construct or variable on the causal pathway from a predictor to an outcome, whereas a moderator affects the relationship between a predictor and outcome (e.g., changes the magnitude of the effect), but does not lie on the causal pathway [73]. In seeking to understand the role of self-compassion in social media, self-compassion was hypothesized to be both a mediator and a moderator throughout the manuscripts reviewed.

##### Mediating and moderating effects of self-compassion in the context of psychological well-being.

Two studies in this review examined self-compassion as a mediator in the context of social media use and well-being [59,64]. The aforementioned Mosanya [64] article uniquely examined self-compassion as a mediator on the pathway from social media addiction to self-esteem, allowing authors to examine the direct effect of social media addiction on self-compassion as well as that of self-compassion on self-esteem. Mosanya and team found a significant inverse relationship for these effects, but above all found that self-compassion mediated the relationship between social media addiction and self-esteem [64]. These mediation results determined that self-compassion served as a considerable buffer to the negative effect of social media addiction on self-esteem for both men and women in this Polish sample. One other study in this review conducted by Keutler and colleagues [59] examined self-compassion as a mediator in the context of well-being among Irish undergraduate students. These researchers found that high perfectionistic presentation on social media predicted lower levels of self-compassion, and that lower levels of self-compassion predicted lower levels of subjective well-being. When self-compassion was considered as a mediator, it buffered the negative effects of perfectionistic self-presentation on social media on subjective well-being. These results suggest self-compassion may present an opportunity for intervention to lessen the harm of disadvantageous social media use.

Six studies examined self-compassion as a moderator in the context of social media use and psychological well-being [41,47,54,57,58,70]. A majority of these studies found self-compassion to be a nonsignificant moderator when examining the direct and indirect effects of social media use and well-being outcomes. Adkins and colleagues [47] found that self-compassion did not moderate the indirect or direct relationship between photo-oriented social media use and stigma among UK adults, while Einstein’s article [54] found that self-compassion was not a significant moderator of the relationship between social media frequency and anxiety among Australian adolescents. Similarly, results from a Netherlands-based cross-sectional survey study [57] concluded that self-compassion did not significantly moderate the effect of social media use on positive affect. In this article, researchers measured social media in a multitude of ways including presence and duration of use, most-used platform, and activity and quality of social media use, but still found no moderating effect. Finally, in a sample of young children from China, Guo and colleagues [58] reported that while self-compassion moderated the indirect effect of social media use on depression by way of self-esteem (moderating both pathways from social media to self-esteem and self-esteem to depression), self-compassion did not moderate the direct effect of use on depression.

Conversely, there were two studies which found self-compassion to be a significant moderator in the context of social media use and well-being. Shao and colleagues [70] found that intrapersonal regulation (e.g., self-kindness) moderated the impact of emotional exhaustion on outcome appraisal among Chinese internet users - that is, self-kindness protected emotionally exhausted individuals from negative or unfavorable outcome reappraisal. However, they also concluded that these benefits may be counteracted by hyper personal regulation (e.g., social media-based emotional regulation). Shao and team posited that lack of context clues during online conversation leads to individuals feeling judged about disclosure of negative sentiment in online spaces, rather than relieved or reassured as they may feel when having these conversations in-person [70]. Finally, while Phillips and colleagues [41] found self-compassion to be a significant moderator on the relationship between social media use and anxiety and depression, it was their innovative and novel measurement of social media use that proved most impactful to the field of social media research. Informed by the five systems model [74], researchers constructed social media profile types using relevant variables including: 1) frequency of social media use, 2) time spent on social media, 3) problematic social media use, 4) perception of social media interactions, 5) emotional responses to social media, and 6) fear of missing out (FoMO). Profile types included 1) Problem Users, 2) Disenchanted Dabblers, 3) Moderate Users, and 4) Contented Dabblers. As hypothesized, higher levels of self-compassion were associated with less time spent on social media, fewer visits to social media platforms, less problematic use, less FoMO, and with more positive online interactions and emotional responses. Finally, in reflection of moderation analyses, the Phillips’ article reported that although Problem Users reported higher mean levels of anxiety and depression than other profile groups, self-compassion buffered this relationship, with highly self-compassionate Problem Users reporting similar anxiety and depression levels to other profile groups.

##### Mediating and moderating effects of self-compassion in the context of appearance/body image.

As with well-being outcomes, the Mosanya article was one of two to examine self-compassion as a mediator in the context of appearance/body image and social media use [64,65]. In addition to a direct and significant effect of social media addiction on self-compassion and self-compassion on body image, Mosanya and team [64] found that self-compassion fully mediated the relationship between social media addiction and body image. Mediation by self-compassion was seen in both men and women in this Polish sample. These results highlight the importance of self-compassion in buffering the negative effects of social media addiction on body image outcomes across genders. In agreement with these findings, Roberts and colleagues [65] found self-compassion to be a significant mediator on the pathway between absence of social media use and both body surveillance and body shame. This quasi-experimental study investigated the impact of a 3-day social media fast on body image outcomes in a group of adolescent girls from the US and Canada. Researchers found that the 3-day fast resulted in a positive effect explained by an increase in self-compassion and self-esteem. These findings indicate that self-compassion may be an especially important and useful buffer against self-objectification and other appearance/body image-related outcomes.

Five studies in this review examined self-compassion as a moderator in the context of social media use and appearance/body image [29,47,60,63,66]. In their cross-sectional survey study based out of the UK, Adkins and colleagues [47] found that self-compassion did not moderate the relationship between photo-oriented social media use and upward appearance comparison. Modica [63] similarly found nonsignificant moderation of self-compassion when examining the association between Facebook appearance comparison and both body esteem and body surveillance outcomes. Similar findings were reported by Lonergan and team [60] when they explored the relationship between social media variables, including photo manipulation and photo investment, and body dissatisfaction - that is, self-compassion did not significantly moderate these associations. Conversely, two studies in our review indicated significant moderating effects by self-compassion. Rutter and colleagues [66] found that while trait self-compassion did not significantly moderate the relationship between face only conditions (posts with makeup, without makeup, or landscape photos) and state self-compassion, trait self-compassion did significantly moderate the relationship when participants were shown body-related posts (body positivity body photos, body positive quotes, fitspiration, or landscape photos). That is, those who viewed fitspiration posts reported significantly lower state self-compassion than the other three conditions, but only among those with low trait self-compassion. High trait self-compassion seemed to protect against a negative change in state self-compassion when body-ideal images were viewed. Lastly, Wang and colleagues [29] also found a significant moderating effect of self-compassion in the indirect relationship between body talk on social network networking sites (SNS) and body dissatisfaction via peer appearance pressure among young Chinese women. Specifically, the relationship between body talk on SNS and peer appearance pressure was significant but only among those who reported low levels of self-compassion. Findings from this cross-sectional study suggest that there may be a potential protective role of self-compassion when it comes to body image concerns.

#### Theme 3: Self-compassion-oriented interventions may promote favorable health outcomes, with social-media-based approaches emerging as a promising avenue.

Findings across reviewed manuscripts demonstrate the efficacy of social media-based self-compassion interventions on improving well-being and appearance/body image outcomes. Throughout these interventions, outcomes examined pre- and post-interventions included: self-compassion [49,61,68,69,71], body satisfaction [48,49,61,68,71], exercise motivation [48], body appreciation [49,61,68,71], appearance comparison [49,68,69], body image [39], weight and appearance dissatisfaction [39], self-criticism [61], social media comparison [61], social appearance anxiety [68], drive for thinness [68], face-related appearance shame [69], anxiety [69], and negative mood [69,71]. A majority of these self-compassion interventions intended to examine changes in a variety of appearance/body image outcomes, with only two including psychological well-being outcomes.

In an aforementioned quasi-experimental study, Arigo and colleagues aimed to examine the differential effects of fitspiration posts paired with fitspiration messaging, self-compassion messaging or no text (image only) by randomizing US college students into one of three exposure groups and having them complete an electronic survey post-exposure [48]. This was the only study that found no significant differences in outcomes of interest including body satisfaction and exercise motivation by exposure group, but also the only intervention study that did not include pre- and post-intervention surveys to assess how outcomes changed pre- and post-stimuli exposure.

Three studies in this review examined the impact of social media stimuli (e.g., Instagram posts, Tiktok videos) on appearance/body image and well-being outcomes, measuring outcomes pre- and post-stimuli exposure to assess change [49,69,71]. Slater and team [71] randomly assigned female undergraduate students in the UK to view Instagram fitspiration images, self-compassion quotes, a combination of both, or appearance-neutral images. They found that those who viewed fitspiration images reported significantly less self-compassion post-exposure than women who viewed appearance-neutral (control) images. Additionally, these researchers found that women who viewed self-compassion quotes on Instagram reported greater body satisfaction, body appreciation, self-compassion, and lower negative mood, compared to women who viewed control images. Finally, those who viewed fitspiration images with self-compassion quotes reported greater body satisfaction, appreciation, and self-compassion, and less negative mood compared to those who viewed fitspiration images only. In a similarly-designed study, Barron and colleagues [49] exposed US undergraduates and a young adult community sample to Instagram posts to determine whether these posts influenced participants’ body image and self-compassion. This study [49] used the same condition groups as Slater et al. (e.g., fitspiration, self-compassion, both, and neutral) [71] to assess differential effects of exposure groups. These effects were assessed in a university sample and then replicated in a sample of young adults from the community. Similar to previous findings, researchers reported that those who viewed fitspiration images experienced lower levels of body satisfaction (community sample only), body appreciation (university sample only), and self-compassion (both samples) post-exposure compared to those who viewed neutral content. Both samples reported lower levels of body satisfaction and appreciation after exposure to fitspiration content compared to self-compassion content. In agreement with the Slater article findings [71], participants in the self-compassion only group reported higher levels of body satisfaction and body appreciation. However, only the community sample reported significant differences in self-compassion comparing the self-compassion only to the other condition groups. Contrary to Slater’s findings [71], Barron and team [49] did not find evidence of a buffering effect when participants viewed fitspiration images with self-compassion quotes. By examining these effects in a community sample and in a sample including men, Barron’s findings [49] further generalizability of the potential positive effects of exposure to self-compassion content on social media. Finally, a third article by Seekis and Kennedy [69] examined the impact of beauty, self-compassion, and travel (control) TikTok content on face-related appearance shame and anxiety, self-compassion, mood, appearance thoughts, and upward appearance comparison among undergraduate women in Australia. This similarly designed study randomly assigned women to one of three condition groups and outcomes were measured pre- and post-exposure to detect change. Findings show that women in the beauty group reported more upward appearance comparison and appearance thoughts relative to the other two groups. Additionally, those in the beauty group experienced lower levels of self-compassion, and higher appearance shame and anxiety and negative mood compared to those who viewed self-compassion or travel content. These findings emphasize that presence of self-compassion content on social media may negate some of the negative effects of appearance-related social media content.

Three additional studies examined the impact of self-compassion intervention (e.g., workshop, writing task) on appearance/body image and well-being outcomes, measuring outcomes pre- and post-intervention to assess change [39,61,68]. These interventions incorporated social media in different ways, with two studies using social media as an avenue for support [61,68] and the third using social media posts as an exposure to assess the impact of the intervention [39]. In a quasi-experimental study conducted by Seekis and colleagues [68], researchers sought to understand the impact of a 50-minute Mindful Self-Compassion workshop on body image outcomes and self-compassion among university women in Australia. Women were assigned to the workshop or a waitlist control and followed for 3 months post-intervention. Participants in the workshop group were linked to a private Facebook group where they were asked to utilize the self-compassion techniques they learned when feeling appearance distress and post about their experiences in the group a few times per week for two weeks. Relative to the control group, findings indicated that those who participated in the self-compassion workshop reported lower upward appearance comparison, social appearance anxiety, body dissatisfaction, and drive for thinness and higher body appreciation and self-compassion. These findings held at 1-month and 3-month follow-up, aside from body dissatisfaction. This study highlights the benefits of teaching self-compassion while also emphasizing the importance of sharing moments of self-compassionate experiences in a supportive environment. Another study employed a 5-week school-based compassionate mind training (CMT), Digital Social Media Adolescent Resilience Training (SMART), to assess the impact of intervention on body dissatisfaction among adolescents in Ireland [61]. Similar to the design seen in the previous study, students were assigned to either the intervention or a control waitlist and followed for three months post intervention. The study found gender differences in outcomes - whereas girls experienced an increase in body appreciation and a decrease in self-criticism, boys exhibited an increase in body satisfaction only. No sustained improvements in self-compassion were observed. These gender differences may hint at differences in how body image and/or self-compassion are experienced by boys and girls. Finally, Gobin and colleagues [39] aimed to determine whether a self-compassion micro-intervention could prevent state weight and appearance dissatisfaction increases after exposure to thin ideal images on Instagram. Women in the US and Canada were randomly assigned to the intervention (self-compassion writing task) or control group (simple sorting task) and then were asked to report their state body dissatisfaction. Afterwards, they were asked to compare themselves to thin ideal images on Instagram before reporting their state body dissatisfaction again. Researchers found that those who were assigned the self-compassion writing task experienced decreased weight dissatisfaction and appearance dissatisfaction that sustained after exposure to Instagram images. Those in the control group experienced the opposite effect - an increase in body concern after viewing the thin ideal images. Although each of these self-compassion interventions present differently, the role of self-compassion training in mitigating the harmful effects of social media use is consistent and apparent.

#### Theme 4: Social media use is measured inconsistently, suggesting a standardized measure for this construct is lacking.

As highlighted by a recent National Academies of Sciences, Engineering, and Medicine report on Assessment of the Impact of Social Media on the Health and Wellbeing of Adolescents and Children [46], there is seemingly “no gold standard” for measuring social media use, leaving gaps in our understanding of the interplay between social media use and wellbeing. The ramifications of these gaps were further uncovered in our review, beginning with the variation and lack of standardized measurement approaches observed, which led to difficulty in comparing effects of self-compassion in the context of social media use across studies. It is important to note that the vast majority of studies included in this review quantified social media use in various ways and used more than one method of measurement. Therefore, more than 30 measurements of social media use across the papers reviewed were observed and compiled.

While several measures examined social media use generally (n = 7), assessing using (yes/no) or having a social media account (e.g., number of platforms [47,71]), many papers included more complex measurements of use. For instance, time spent on social media was quantified in two ways: duration and frequency of use. Ten distinct measures examined duration of use. Some studies examined duration using a single item (e.g., time spent on Facebook [63]) while others used multiple items to capture duration (e.g., time spent actively on all social media sites in the past week [29]). Five measures captured an alternative component of time spent – i.e., frequency of use - to quantify social media use, including items such as, ‘Frequency of checking social media’ [50].

Other studies in our review looked beyond time spent on social media use to better understand the reasons for, attitudes towards, and effects of use. Reasons for social media use encompassed both social media interactions and activities. Ten measures quantified a person’s activities on platforms including what a participant was doing on social media and with whom they were interacting. These included validated scales such as the Facebook Questionnaire [47,63,75], Facebook Intensity Scale [58,76], and the Social Media Use and Gratification Scale [67,77]. Three studies used three distinct measures of attitudes toward use including items such as a subjective rating of quality of use [57]. To measure the effect of social media use, four measures of effect were identified in this review. These included items to quantify a respondent’s emotional response to social media [41] while also including validated scales such as the Self-Worth Dependent on Social Media Scale [67,78].

There were other social media use measures that also sought after emotional experiences related to use in the realm of appearance and self-presentation (n = 4). These measures attempted to quantify the value users placed on their social media image and included adapted measures such as the Perfectionist Self-Presentation Scale [59,79] and the Photo Manipulation and Investment Scales [60,80]. It is important to note that three of the four measures that assessed social media self-presentation and appearance were created for real-world context and adapted to a social media context for their respective papers.

Finally, while a majority of these measures imply ‘regular’ or ‘ordinary’ social media use, several studies chose to represent problematic use of social media. Three measures quantified problematic use with validated scales including the Internet Severity and Activities Addiction Questionnaire [52,81], the Smartphone Addiction Scale [55,82], and the Bergen Social Media Addiction Scale [41,62,64,83]. These discrepancies in social media use measurement highlight lack of standardization in the field, leading to inconsistencies across studies and challenges in assessing social media’s relationship with self-compassion, appearance/body image, and well-being.

## Discussion

### Interpretation of results

The purpose of this review was to provide the first detailed overview of existing literature on the association between self-compassion and adverse consequences of social media use on various wellbeing characteristics. Our review was the first, to our knowledge, to examine the associations between these constructs in a wide variety of study designs to better understand potential patterns. While the studies included in this review showed mixed results, particularly across the dimensions of wellbeing and body image related outcomes, we observed some notable consistencies and findings.

First, a number of studies demonstrated similar associations between self-compassion, social media usage patterns, and wellbeing measures. Several studies examined and reported associations between higher levels of self-compassion and lower levels of social media use, lower levels of self-compassion and poorer psychological wellbeing, and higher levels of social media use and poorer psychological well-being. Given that the vast majority of studies were cross-sectional, these associations were suggested to apply in both directions (i.e., higher self-compassion was generally associated with more adaptive wellbeing outcomes). Prior studies have looked at these associations separately, producing similar results across body image and non-body image wellbeing outcomes. In particular, higher levels of self-compassion have been associated with lower levels of psychopathology among adults (e.g., suicidal ideation, self-harm, depressive symptoms, anxiety, post-traumatic stress disorder symptoms, and stress) [17,19,84] and younger populations [14,20]. Additionally, a prior review conducted in 2016 concluded that higher self-compassion was associated with lower body image concerns and eating pathology [85]. Perhaps the least surprising association observed across reviewed studies was the relationship between social media use and various wellbeing outcomes, given the extensive literature that has demonstrated higher social media usage patterns associated with poorer wellbeing outcomes. For example, higher frequency and/or intensity of social media use has been associated with harmful impacts on wellbeing such as, disturbance of body image ideals, increased anxiety, increased depression, and increased suicide risk [28,29]. These patterns have been largely replicated in studies conducted in both adults [86] and youth [87,88]. While no prior study has conducted a systematic or scoping review of the literature examining associations between self-compassion and social media use, one review and meta-analysis conducted in 2019 concluded that self-compassion had a significant impact on self-regulation and health behaviors [89], which may potentially apply to social media usage contexts as social media may constitute or contribute to health risk behaviors [33].

A second major finding from this work is that the reviewed studies unsurprisingly suggest a possible protective role for self-compassion against adverse effects of social media use, but the extent and mechanisms involved are still unclear due to varied findings across studies and potential limitations in study design and measurement, which we explore in further detail below. With no prior reviews conducted examining these constructs, it is difficult to identify these specific mechanisms without further exploration.

Third, reviewed studies showcase the effectiveness of self-compassion interventions in increasing self-compassion with social media-based interventions as a promising option. However, the mechanics of how these interventions are delivered via social media may impact the longevity and strength of effects. Interestingly, studies included in our review largely focused on outcomes related to body image and eating pathology. While no prior studies have established a baseline of effectiveness for these types of interventions, prior reviews have examined aspects aligned with our broader findings. For example, Ferrari et al.’s [90] meta-analysis of the efficacy of self-compassion-oriented interventions in randomized controlled trials (RCTs) on various psychosocial outcomes found that these self-compassion interventions led to significant improvement across 8 different areas compared to controls. The areas with the largest effect size (Hedge’s g) were eating behavior (g = 1.76) and rumination (g = 1.37), with moderate effects for self-compassion, stress, depression, mindfulness, self-criticism, and anxiety outcomes. Similarly, a review and meta-analysis conducted in 2022 [91] investigated the overall effect of self-compassion-related interventions on self-criticism and concluded that self-compassion-related interventions significantly reduced self-criticism in comparison with control groups. Their study also found that length of the intervention was a significant moderator in the reduction of self-criticism, suggesting that the length of interventions plays a significant role. This is notable because self-criticism has been identified as having positive associations with depressive symptoms, symptoms of eating disorders, social anxiety disorder, personality disorders, and psychotic symptoms or interpersonal problems [92]. Additionally, a scoping review conducted in 2022 [93] is the only one to our knowledge that has examined use of online programs and mobile applications of self-compassion, mindfulness, and meditation by workers. However, their review did not examine effectiveness of these interventions and only provided a preliminary landscape of the literature and recommendations for future studies in order to better understand the contexts and individuals for whom these interventions are most effective [93].

Finally, our review put in stark view the current lack of rigorous and standardized social media measurement throughout the literature. Specifically, a key takeaway from this work is that currently, there is no “gold-standard” for measuring social media use, and lack of standardization in measurement approaches may limit understanding of the impacts of social media usage on wellbeing and self-compassion. Furthermore, prior work has also highlighted the difficulty in accurately measuring social media use due to its complexity as a health behavior and as a construct to appropriately conceptualize and operationalize. For example, as a 2020 review highlights, there have been 25 distinct theories or models used to guide research in the realm of studying problematic social media use behaviors spanning from individual level mechanisms to organizational and systemic level factors [94]. This wide range in how social media use may be conceptualized and operationalized and lack of agreement on which approach most appropriately corresponds with health and wellbeing outcomes, contributes to these existing gaps in measurement. Different measurement tools or mismatched conceptualization and operationalization of approaches may lead to variations in results across studies, potentially obscuring the true nature of associations between these constructs. Taken together, our review revealed a promising role for self-compassion in promoting wellbeing and buffering against the adverse effects of social media usage but also revealed several gaps in this area of research and highlighted this as a future direction.

### Limitations of included studies

In exploring the landscape of the literature, we observed an immaturity in this field. Our findings suggest that this may be due to limitations in study design and methodology and in the varied approaches to measurement of relevant constructs. Within these categories, we expand on key concerns regarding limitations due to reliance on cross-sectional study design, questions about individual vulnerability that have yet to be addressed or understood in the context of social media usage and exposures, and lack of uniform measurement of social media (and reliance on self-report). Future research would benefit from thoughtful consideration for how study designs may be innovated to address these current limitations of the existing literature.

#### Study design and methodology.

First, mixed findings across studies included in this review may be attributed to various issues related to the study design and sample characteristics. The reviewed articles largely looked at self-compassion as a moderator or mediator in the association between social media use and various wellbeing characteristics (mainly related to psychopathology and body image constructs), with various limitations restricting our knowledge of how, when, and in whom fostering self-compassion may be most beneficial. Most of the studies included in this review were cross-sectional and conducted with relatively small and homogeneous samples where individual variations related to social determinants, vulnerability, emotion regulation, and resilience were not measured or evaluated. Mixed findings may have also been related to sample-specific issues in the reviewed studies, such as power limitations due to small effect sizes and insufficient sample sizes.

Methodologically, cross-sectional studies are limited in their ability to comment on temporality and causality as it may relate to how self-compassion and social media usage are associated [95,96]. While many studies included in the review reinforced an association between high levels of self-compassion and less problematic or frequent use of social media, cross-sectional designs do not allow for conclusions regarding the directionality of and degree of causality in these relationships. Based on existing literature, it is unclear if self-compassion levels are responsible for social media usage patterns (i.e., high self-compassion is responsible for less problematic social media usage patterns) or if social media usage patterns are responsible for self-compassion levels (i.e., less problematic or frequent social media usage is responsible for higher levels of self-compassion). Studies conducted using designs able to establish and evaluate temporality and causality (i.e., cohort studies, randomized controlled trials, quasi-experimental studies [95,96]) are necessary to comment further on the mechanics of how self-compassion and social media usage may impact one another. Prior studies that have aimed to longitudinally examine relationships between social media use and wellbeing have not examined self-compassion, opting for constructs such as self-esteem [97], despite the collective understanding that self-compassion may be a more balanced characteristic in ensuring well-being in the context of risks posed by internet usage [98].

Additionally, a lack of sample diversity may have limited the ability to determine the true magnitude of protective effects of self-compassion in understudied populations. The lack of diversity in samples also may have concealed alternate mechanisms by which these effects may operate, especially within populations underrepresented in this review. Articles eligible to be included in our review largely consisted of all female, majority white, and overwhelmingly young (university-based) samples. Half of the articles (n = 15) did not report on racial identity and all 30 articles failed to measure additional characteristics such as language, socioeconomic status, social support, and mental health status that may independently operate as social determinants of wellbeing [99]. Furthermore, none of these studies collected information regarding participants’ experiences of discrimination or structural barriers that may impact their mental health and wellbeing, despite prior work establishing the importance of understanding individuals’ experiences of discrimination in this context of wellbeing [100–103] and internet usage patterns [104]. Only about half of the reviewed samples included participants who identified their gender as male, restricting researchers from examining gender differences in the association between social media use and self-compassion. Only two studies [61,64] examined and found gender differences in this association. It is also possible that self-compassion may not provide a significant protective benefit in certain circumstances related to social media use, and interindividual differences should be explored further to better understand how self-compassion operates across varied populations and contexts. However, it is difficult to identify relevant explanatory factors without adequate sample diversity and measurement of additional relevant constructs. As such, there are gaps in our understanding, including that we do not yet have a cohesive model of what factors play into these associations, including how variables outside of online behaviors and activity may impact these associations.

#### Measurement of relevant constructs.

Issues of measurement of constructs may also have contributed to limitations. Notably, one question that arose in the process is whether measurement of self-compassion was necessarily consistent and accurate across studies. For example, while the Rutter et al study [66] used Neff’s Self-Compassion Scale [4] in their intervention study to measure trait self-compassion as a potential moderator, they used an adapted version [105] of the state Self-Compassion measure [12], developed by Neff et al to aid in measurement of changes in self-compassion due to intervention. Use of measurement tools developed for a particular study that have not been validated create difficulty when evaluating results uniformly and in the context of comparison to results observed in other studies.

Also noteworthy, within our review, studies on body image yielded less conclusive outcomes (compared to studies with more psychopathology focused wellbeing outcomes). This may be due to the multidimensional nature of body image as a construct, as well as the diverse methods used to measure aspects or subdimensions of body image across studies. Studies focused on body image related outcomes in our review did not incorporate measures of well-being or mental health, limiting the ability to contextualize findings. This is particularly salient in cases where clinical conditions or symptoms could significantly influence an individual’s body image or sense of self or where disturbances in body image may negatively influence development or exacerbation of clinical conditions or symptoms. As such, measurement of body image constructs in these studies may not be adequate to accurately characterize the intensity or severity of mental health concerns participants may be managing. The lack of such measures in the reviewed studies make it challenging to control for variability within participant groups.

Findings from this review also imply the need for a more rigorous and standardized approach to measurement of social media use [41] that targets multiple social media platforms [40] and taps into specific mechanisms of negative online behaviors [59]. In addition, most studies employed coarse and imprecise measures of social media use that largely rely on self-report and are potentially vulnerable to recall bias [106,107]. The literature makes apparent that the method of measurement impacts what can be studied and what conclusions can be drawn, with some measurement approaches offering more useful insights than others due to the granularity and transparency of what is being measured and how it may operate [25,42,108].

Along these lines, there were variations in findings across studies included in our review that examined self-compassion as a moderator between social media usage and depression and anxiety. For example, several studies found that self-compassion did not significantly moderate the relationship between social media usage and anxiety [54] or depression [58], however the Phillips et al article [41] that constructed social media use profiles found a significant moderation effect of self-compassion on anxiety and depression. Inconsistencies across findings from these studies may be attributable to a variety of reasons. For one, these differences may be due to variation in study design ranging from study specific factors such as study sample characteristics (Guo et al.’s [58] sample was comprised of children versus Phillips et al.’s [41] comprised of adults), differences in social media measurement approaches (Phillips et al [41] designed a specific characterization for social media use in the context of wellbeing, versus Einstein et al [54] and Guo et al [58] who respectively used more simplistic approaches, a single frequency item and an adapted 8 item Facebook Intensity Scale originally validated in adults [76]), or true differences in the study populations or mechanisms of self-compassion.

Additionally, while some validated scales have been developed to measure problematic social media usage behaviors, there is no universal understanding or approach to evaluate beneficial, positive, or neutral social media usage behaviors. This results in a default assumption that low intensity or low frequency usage of social media is always associated with better health and wellbeing outcomes. However, while this direction of association may be characteristic of many of the results of this review and other broader studies examining these constructs, it is important to note that there may be specific social media behaviors or usage patterns associated with positive health and wellbeing outcomes that we are unable to observe with existing tools and approaches. Prior reviews have highlighted the existence of this range in social media usage that may be associated with positive health outcomes or at least the absence of negative health outcomes [86–88]. Existing scales and measurement of social media use may not be calibrated to capture potentially beneficial online behaviors or usage patterns due to a lack of granularity and complexity.

Separately, while we limited our review to quantitative studies only, qualitative research holds unique value in being able to explore and better understand the associations examined in this review and help address these limitations articulated in this discussion section. One study in particular [51] highlighted the importance of mixed methods approaches to better understand and interpret results from quantitative cross-sectional data, and to more accurately explore themes related to sample composition and measurement issues. Results from the qualitative portion of this study [51] showed that participants used social media as a medium for venting their feelings, to share negative experiences, and receive support from others with similar experiences. These findings [51] are particularly notable considering the study’s sociocultural context, as the sample was composed of undergraduate students in Thailand. It has been hypothesized that collectivist cultural norms in Thailand may impact behaviors and internalization of certain social expectations. This could potentially explain the feelings of guilt or shame associated with choosing to use social media rather than engaging with family or friends. Though students said that they can find emotional support online, it was revealed that this support may not be seen as superior to face-to-face support. Some students also reported using social media to avoid unpleasant emotions or feelings, revealing potentially maladaptive emotional coping mechanisms entangled with technology use. Ultimately, this study [51] establishes the existence of this association and other relevant factors when understanding the interplay of social media use, self-compassion, and wellbeing. Specifically, this highlights how beliefs about and implications of certain patterns of social media use, in addition to exposure to potentially harmful content on these platforms, may contribute to lower levels of self-compassion or self-regard. Despite this study’s limitations of limited sample and temporality, it warrants further research exploring this association in broader populations and with more specific measures, while also emphasizing the value of qualitative exploration in this area.

### Limitations of the review

There are a few limitations that may help contextualize the interpretation of findings presented in our scoping review. First, we excluded grey literature (dissertations, theses, conference abstracts, white papers, unpublished manuscripts, etc.) and clinical trial registry records in our search. This was done to streamline our ability to scope the landscape of published literature as the first scoping review exploring these constructs. However, by excluding unpublished studies from our review, there is potential for publication bias [109]. Second, we limited our search to only include studies published or available in English. Despite the limitation to English publications, studies included in our review were conducted in countries across the world, including China, Greece, Iran, the Netherlands, Poland, Spain, and Thailand. Finally, our review was limited to only quantitative studies which was intended to streamline this initial scoping process for examining patterns across the constructs of social media, self-compassion, and wellbeing. While this may have been appropriate for the initial examination of the literature, this excluded inclusion of qualitative insights regarding the associations between self-compassion, social media, and wellbeing that may have added nuance or more granular explanation of observed associations. Future reviews on this topic may benefit from considering examination of unpublished studies and grey literature, studies published in languages other than English, and studies utilizing mixed methods or purely qualitative designs in order to evaluate more nuanced insights about the nature of these associations.

### Implications of the results for practice and future research

In conclusion, this review highlights the potential of self-compassion to enhance wellbeing and mitigate the negative impacts of social media use, and we note four specific areas that emerged as priorities for future research and practice.

First, more sophisticated and precise measurements of social media use (why people use social media, how they use it, what they do while using it, how dependent they are on it, what emotional connection they have with it) are needed to properly assess the association of use on wellbeing outcomes. The field would benefit from research that works towards developing, validating, and using measures that aim to capture these themes of usage more distinctly.

Second, studies that incorporate additional context on individuals’ lives and sources of individual-level variation in vulnerability and resilience to adverse impacts of social media exposure are also needed. Doing so may elicit more useful insights about patterns in usage, wellbeing, and opportunities where self-compassion may provide buffering. Individual-level factors should include social determinants of health, as well as aspects of emotional processing, coping, and regulation that may impact how an individual uses social media.

Third, from a clinical standpoint, measurement of constructs (self-compassion, social media usage, and wellbeing outcomes) and incorporation of self-compassion interventions into evidence-based therapeutic practice may benefit clients, especially those who may be vulnerable to distress or harms posed by problematic social media use. Adaptation of existing self-compassion-based interventions to emphasize reduction in self-critical tendencies and enhance mindfulness. This may be especially beneficial for building resilience and fostering emotion regulation skills in both youth and adults.

Fourth, incorporation of digital literacy education with self-compassion concepts targeted to youth and parents may offer a preventative approach to equip youth with tools to more safely navigate online spaces. An integrated curriculum such as this may emphasize mindfulness skills, to help support better emotion regulation and awareness of social media usage patterns (i.e., to be able to identify if and when social media is being used as a coping mechanism or distraction). Incorporation of education regarding algorithms, veracity of information and content presented online, as well as digital safety practices is also critical for ensuring youth are prepared to navigate these online spaces. This curriculum may also benefit from including suggestions for how youth may use social media platforms to boost their wellbeing, for example, emphasizing the value of sharing and consuming self-compassionate content and engaging in online communities.

## Supporting Information

S1 Checklist Preferred Reporting Items for Systematic reviews and Meta-Analyses extension for Scoping Reviews (PRISMA-ScR) Checklist. (DOCX)

S1 Table Search Strategies. (DOCX)
