# Supplementary material for: Self-compassion as a protective factor against adverse consequences of social media use: A scoping review
Source: PLoS One. 2025 May 21;20(5):e0322227. doi: 10.1371/journal.pone.0322227 (PMC12094758; doi:10.1371/journal.pone.0322227)
Supplement: S1 Table — (DOCX) [file pone.0322227.s002.docx]

S1 Table. Search Strategies

*Reported results are records obtained from the search conducted March 2023 to June 2023 conducted by two reviewers using Google Scholar, PubMed, PsycInfo, and PsycArticle databases. Reported records represent raw results without duplicates or ineligible records removed.

**Reported results are records obtained from the follow-up update search conducted in October 2024 limited to any papers published in 2023-2024 conducted by two reviewers using Google Scholar, PubMed, PsycInfo, and PsycArticle databases. Reported records represent raw results without duplicates or ineligible records removed. The abstract and titles of each of these updated records were reviewed.

Google Scholar

| **Search** | **Terms** | **Records** | **Records (10/4/24 search)**** |
| --- | --- | --- | --- |
| 1 | “Self compassion” AND “social media use” | 11 | 15 |
| 2 | "social media use" OR "social media usage" OR "social media profile" AND "self-compassion" OR "self compassion" OR "self regard" | 2220 | 1026 |

PubMed

| **Search** | **Terms** | **Records** | **Records (10/4 search)**** |
| --- | --- | --- | --- |
| 1 | (social media) AND (self-compassion) | 59 | 43 |
| 2 | ((social media use) OR (social media usage) OR (social media profile)) AND ((self-compassion) OR (self compassion) OR (self regard)) | 315 | 84 |

APA PsycInfo

| **Search** | **Terms** | **Records** | **Records (10/04 search)**** |
| --- | --- | --- | --- |
| 1 | "social media use" AND "self-compassion" OR "self compassion" | 4 | 7 |
| 2 | "self-compassion" OR "self compassion" AND "social media use" OR "social media" | 37 | 22 |
| 3 | "social media use" OR "social media usage" OR "social media profile" AND "self-compassion" OR "self compassion" OR "self regard" | 55 | 9 |

APA PsycArticles

| **Search** | **Terms** | **Records** | **Records (10/04 search)**** |
| --- | --- | --- | --- |
| 1 | "social media use" AND "self-compassion" OR "self compassion" | 3 | 2 |
| 2 | "self-compassion" OR "self compassion" AND "social media" OR “social media use” | 2 | 2 |
| 3 | "social media use" OR "social media usage" OR "social media profile" AND "self-compassion" OR "self compassion" OR "self regard" | 4 | 2 |

Search 1:

Total texts reviewed: 2710

Remaining texts after duplicates and non peer-reviewed articles detected and removed in initial screening: 2679

Search 2 (October 2024 Update); search starting in 2023

Total texts reviewed: 1212

Remaining texts after duplicates and non peer-reviewed articles detected and removed in initial screening: 889
